# Rheumatoid arthritis and chronic obstructive pulmonary disease in US adults: a cross-sectional analysis

**DOI:** 10.3389/fmed.2025.1577180

**Published:** 2025-05-23

**Authors:** Panpan Mi, Huijie Wang, Guofeng Fan, Shengle Chen, Xiaoyuan Chen, Xu Cao, Haixia Feng

**Affiliations:** ^1^Department of Orthopedics, Hebei Petro China Central Hospital, Langfang, China; ^2^Department of Endoscopy, Shijiazhuang Traditional Chinese Medicine Hospital, Shijiazhuang, China; ^3^Department of Tuberculosis, Shandong Public Health Clinical Center, Shandong University, Shandong, China

**Keywords:** rheumatoid arthritis, chronic obstructive pulmonary disease, National Health and Nutrition Examination Survey, cross-sectional study, association analysis

## Abstract

**Background:**

Globally, chronic obstructive pulmonary disease (COPD) has emerged as one of the most prevalent chronic respiratory disorders, imposing substantial healthcare challenges and contributing significantly to premature mortality. Emerging evidence indicates that chronic inflammation and immune responses play crucial roles in the pathogenesis and progression of COPD. Rheumatoid arthritis (RA) is a chronic immune-mediated multisystem disease, but its association with COPD remains inconsistent. The primary objective of this investigation is to assess the potential association between RA and COPD.

**Methods:**

Data were derived from the National Health and Nutrition Examination Survey from 2007 to March 2020, which encompassed 25,682 participants. Multivariable logistic regression models were employed to assess the relationship between RA and COPD. To comprehensively evaluate the association’s robustness, we conducted subgroup analyses along with sensitivity analyses, examining potential confounding factors and effect modifiers.

**Results:**

RA was associated with a higher prevalence of COPD even after adjusting for demographic, socioeconomic, lifestyle, and health-related factors (adjusted OR = 1.52, 95% CI: 1.23–1.87, *p* < 0.001). Sensitivity and subgroup analyses confirmed the robustness of these results.

**Conclusion:**

RA is significantly associated with COPD in US adults, highlighting the importance of early detection and preventive strategies aimed at mitigating COPD risk in patients with RA.

## 1 Introduction

Chronic obstructive pulmonary disease (COPD) is a major global health concern, with its prevalence on the rise (1). Although smoking is the main risk factor for COPD development and progression (2), the significant number of non-smoking COPD patients indicates the involvement of additional factors (3). Emerging evidence indicates that chronic inflammation and immune responses are key contributors to the development and advancement of COPD (4–6) and have also shown associations with autoimmune diseases such as rheumatoid arthritis (RA) (7–9).

RA is a chronic immune-mediated multisystem disease, the most common systemic inflammatory rheumatic disease (10, 11). Patients with RA often have extra-articular manifestations, including pulmonary involvement, manifesting as airway or parenchymal inflammation and fibrosis (12). Indeed, up to half of patients with RA develop respiratory diseases over their lifetime (13), and previous research has demonstrated a higher incidence of COPD in patients with RA compared with those without RA (14). These observations suggest a shared pathophysiological basis—encompassing systemic inflammation, smoking, and dysregulated immune responses (7). Notably, seropositive RA is characterized by the production of anti-citrullinated protein antibodies, which can form immune complexes in the lung, activate macrophages, and trigger proinflammatory cytokine release, potentially damaging lung tissue (15–17). Additionally, smoking is regarded as a major shared risk factor in the development of both RA and COPD, possibly heightening COPD risk in patients with RA via oxidative stress and immune dysregulation (18).

Despite these recognized mechanistic overlaps, findings on the RA-COPD relationship have been inconsistent across different populations (8, 19–23). Moreover, there is no nationally representative data examining whether this association holds among U.S. adults. Therefore, our study, using data from the National Health and Nutrition Examination Survey (NHANES), aims to test the hypothesis that RA is independently associated with COPD and to evaluate potential effect modifiers of this association. Elucidating these relationships can aid healthcare providers in detecting COPD and arthritis symptoms earlier and inform more effective preventive strategies.

## 2 Materials and methods

### 2.1 Study population

The data for this study are derived from the NHANES conducted by the Centers for Disease Control and Prevention. NHANES is a nationally representative, cross-sectional survey designed to evaluate the health and nutritional status of the U.S. civilian non-institutionalized population through interviews, physical examinations, and laboratory tests. This nationwide representative survey collects information including demographic data, physical measurements, and laboratory data from participants. The NCHS Research Ethics Review Board approved all survey protocols, and all participants provided written informed consent.

**Figure 1:**
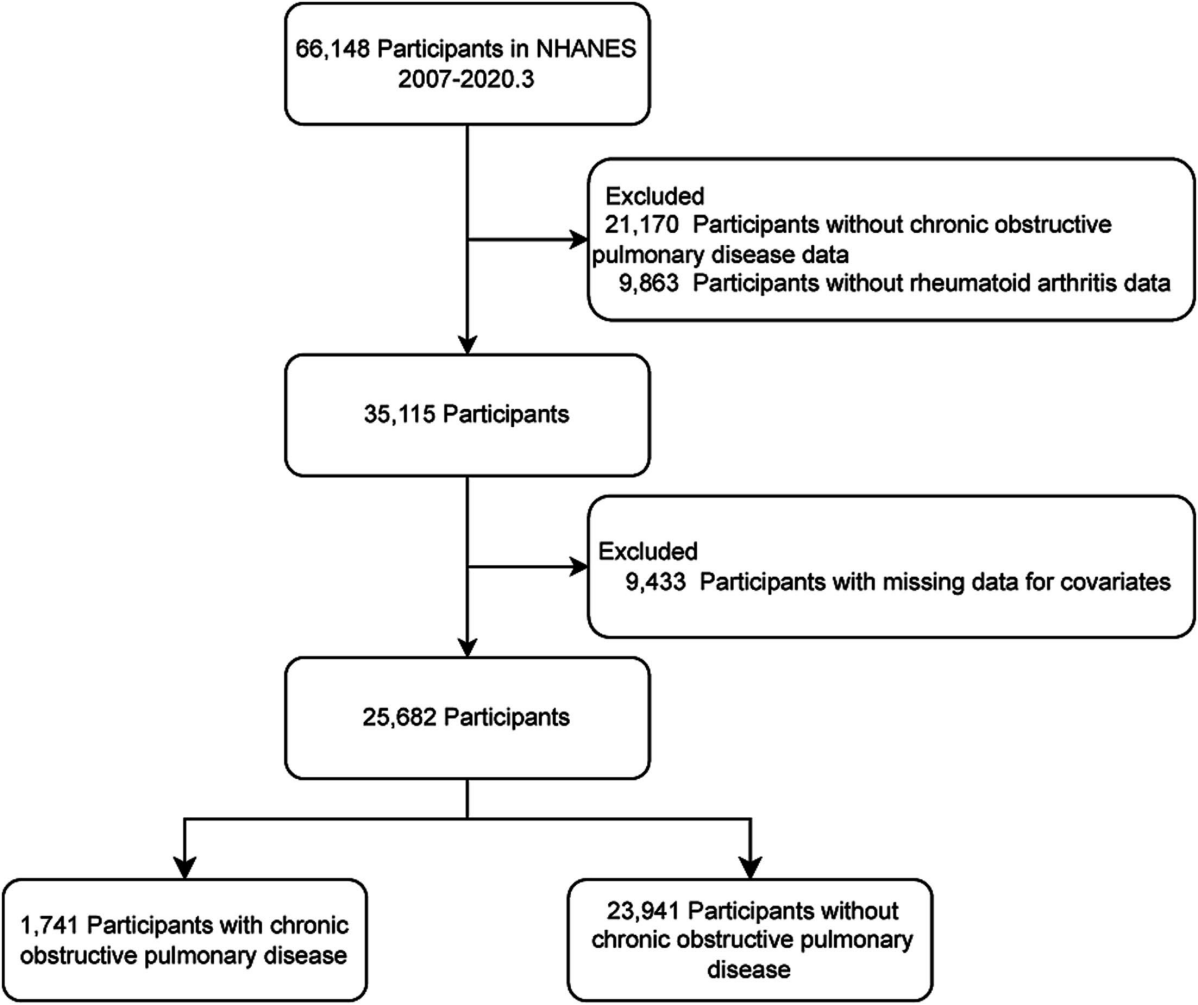
The flow chart.

We selected the NHANES cycles that include information on RA and COPD, covering the survey periods from 2007 to March 2020. Initially, 66,148 participants were included. We excluded those without COPD data (*n* = 21,170), without RA data (*n* = 9,863), and those with missing covariate data (*n* = 9,433). Finally, 25,682 participants were analyzed (for details, see Figure 1).

### 2.2 COPD

COPD diagnosis was established using objective spirometry criteria and self-reported medical diagnoses (24). Trained technicians conducted spirometry in adherence to guidelines set by the European Respiratory Society and the American Thoracic Society. Airflow limitation was defined by a post-bronchodilator forced expiratory volume in one second to forced vital capacity ratio of less than 0.70. Additionally, participants were classified as having COPD if they reported being diagnosed with emphysema, chronic bronchitis, or COPD by a physician or healthcare professional.

### 2.3 RA

The diagnosis of RA in this study was determined using a self-reported questionnaire. Participants were initially asked whether a healthcare professional had ever informed them of an arthritis diagnosis. Those who affirmed this were subsequently questioned about the specific type of arthritis diagnosed. Based on these responses, participants were categorized into either the RA group or the non-RA group. Previous research has validated the acceptability of self-reported RA diagnoses for large-scale epidemiological studies, particularly when a direct rheumatologist examination is impractical (25).

### 2.4 Covariates

In this study, we adjusted for multiple covariates to ensure the accuracy of the assessment of the association between RA and COPD. These covariates included: age, sex, body mass index (BMI), race, education level, marital status, poverty income ratio, physical activity, smoking status, drinking status, high-density lipoprotein cholesterol (HDL-C), total cholesterol (TC), systemic immune inflammation index (SII, platelet count × neutrophil count/lymphocyte count), hypertension, diabetes, coronary heart disease, and stroke.

### 2.5 Statistical analysis

All analyses were performed using R (v.4.2.2) and Free Statistics software (v.2.1), incorporating the sample weights, with results reported as odds ratios (ORs) and 95% confidence intervals (CIs). Baseline characteristics were compared using the Student’s *t*-test (continuous variables) and the chi-square test (categorical variables). To address missing data, multiple imputation by chained equations was applied. The association between RA and COPD was assessed using multivariable logistic regression and subgroup analyses, with confounders selected based on literature or a >10% change-in-effect estimate. Four models were evaluated: an unadjusted Crude Model, Model 1 (adjusted for sex and age), Model 2 (further adjusted for BMI, race, smoking status, drinking status, marital status, education level, physical activity, and poverty income ratio), and Model 3 (additionally adjusted for TC, HDL-C, and SII). Statistical significance was defined as a *p*-value <0.05.

## 3 Results

This study analyzed data from 25,682 participants, including 1,741 individuals with COPD and 23,941 without COPD (Table 1). The results indicated that the COPD group had a higher mean age, greater BMI, lower educational attainment, lower poverty income ratio, and a higher proportion of insufficient physical activity and smoking. Additionally, the COPD group exhibited a higher prevalence of RA, coronary heart disease, diabetes, hypertension, and stroke. Significant differences were also observed in racial and marital status distributions. These findings suggest that COPD is significantly associated with various demographic, socioeconomic, lifestyle, and health-related factors.

**Table 1:** Participants characteristics, weighted.

Variables	Total *n* = 25,682	With COPD *n* = 1,741	Without COPD *n* = 23,941	*p*-value
Age, year	46.76 (0.26)	57.28 (0.57)	46.02 (0.25)	<0.001
Sex, male	12,718 (48.98%)	897 (47.47%)	11,821 (49.08%)	0.320
BMI, kg/m^2^	29.09 (0.08)	30.19 (0.24)	29.02 (0.09)	<0.001
Marital status				<0.001
Married or living with a partner	15,414 (63.86%)	927 (59.51%)	14,487 (64.16%)	
Widowed/divorced/separated	5,380 (17.44%)	598 (28.94%)	4,782 (16.64%)	
Never married	4,888 (18.70%)	216 (11.55%)	4,672 (19.20%)	
Race				<0.001
Mexican American	3,697 (8.26%)	95 (2.51%)	3,602 (8.66%)	
Other Hispanic	2,623 (5.76%)	132 (3.52%)	2,491 (5.91%)	
Non-Hispanic White	10,977 (68.27%)	1,063 (78.50%)	9,914 (67.56%)	
Non-Hispanic Black	5,319 (10.06%)	312 (8.20%)	5,007 (10.19%)	
Others	3,066 (7.65%)	139 (7.26%)	2,927 (7.68%)	
Education level, year				<0.001
<9	5,491 (13.67%)	443 (17.83%)	5,048 (13.38%)	
9–12	5,809 (22.85%)	459 (30.46%)	5,350 (22.32%)	
>12	14,382 (63.48%)	839 (51.71%)	13,543 (64.30%)	
Poverty income ratio	3.09 (1.54, 5.00)	2.26 (1.22, 4.28)	3.14 (1.57, 5.00)	<0.001
Physical activities				<0.001
Inadequate	14,667 (62.67%)	814 (53.20%)	13,853 (63.34%)	
Adequate	11,015 (37.33%)	927 (46.80%)	10,088 (36.66%)	
Smoking status				<0.001
Never	14,468 (56.40%)	478 (27.05%)	13,990 (58.46%)	
Former	6,064 (24.66%)	626 (36.17%)	5,438 (23.86%)	
Current	5,150 (18.93%)	637 (36.78%)	4,513 (17.68%)	
Drinking status				<0.001
No	3,299 (9.80%)	142 (6.27%)	3,157 (10.05%)	
Yes	22,383 (90.20%)	1,599 (93.73%)	20,784 (89.95%)	
HDL-C, mg/dL	53.53 (0.23)	52.62 (0.60)	53.60 (0.24)	0.012
TC, mg/dL	192.49 (0.50)	192.77 (1.56)	192.48 (0.51)	0.740
SII	535.10 (3.83)	618.11 (12.49)	529.30 (3.68)	<0.001
RA	1,501 (4.27%)	221 (9.89%)	1,280 (3.87%)	<0.001
Hypertension	10,490 (36.15%)	1,062 (56.16%)	9,428 (34.75%)	<0.001
Diabetes	4,624 (13.56%)	496 (23.49%)	4,128 (12.87%)	<0.001
Coronary heart disease	974 (3.28%)	221 (12.45%)	753 (2.64%)	<0.001
Stroke	897 (2.71%)	152 (8.47%)	745 (2.30%)	<0.001

COPD, chronic obstructive pulmonary disease; BMI, body mass index; TC, total cholesterol; HDL-C, high-density lipoprotein cholesterol; SII, systemic immune inflammation index; RA, rheumatoid arthritis. Categorical variables are expressed as unweighted counts (weighted percentages), while continuous variables are reported as weighted means (standard deviations) or weighted medians (interquartile ranges).

The multivariable logistic regression analysis (Table 2) demonstrated a significant association between RA and COPD. In the crude model, RA was significantly associated with COPD (OR = 2.72, 95% CI: 2.26–3.29, *p* < 0.001). After adjusting for sex and age in Model 1, the association remained significant (OR = 1.94, 95% CI: 1.59–2.37, *p* < 0.001). Further adjustments in Model 2, which included demographic, socioeconomic, lifestyle, and health-related factors, still showed a significant association (OR = 1.54, 95% CI: 1.25–1.90, *p* < 0.001). Finally, in Model 3, which included additional adjustments for TC, HDL-C, and SII, the association between RA and COPD remained significant (OR = 1.52, 95% CI: 1.23–1.87, *p* < 0.001). Clinically, this adjusted OR indicates approximately 52% higher odds of COPD among patients with RA compared with individuals without RA.

**Table 2:** Multivariable logistic regression analysis of the association between rheumatoid arthritis and chronic obstructive pulmonary disease, weighted.

Model	OR (95% CI)	*p*-value
Crude Model	2.72 (2.26–3.29)	<0.001
Model 1^a^	1.94 (1.59–2.37)	<0.001
Model 2^b^	1.54 (1.25–1.90)	<0.001
Model 3^c^	1.52 (1.23–1.87)	<0.001

a Adjusted by sex and age.

b Adjusted by Model 1 + body mass index, race, smoking status, drinking status, marital status, education level, physical activity, poverty income ratio, diabetes, coronary heart disease, stroke, and hypertension.

c Adjusted by Model 2 + total cholesterol, systemic immune inflammation index, high-density lipoprotein cholesterol.

Sensitivity analysis (Supplementary Table S1) using multiple imputations confirmed the robustness of these results. The crude model in the imputed data showed an OR of 2.93 (95% CI: 2.42–3.55, *p* < 0.001). The association remained significant after adjusting for sex and age (OR = 2.06, 95% CI: 1.69–2.50, *p* < 0.001), further covariates in Model 2 (OR = 1.66, 95% CI: 1.35–2.03, *p* < 0.001), and additional adjustments in Model 3 (OR = 1.64, 95% CI: 1.34–2.01, *p* < 0.001).

**Figure 2:**
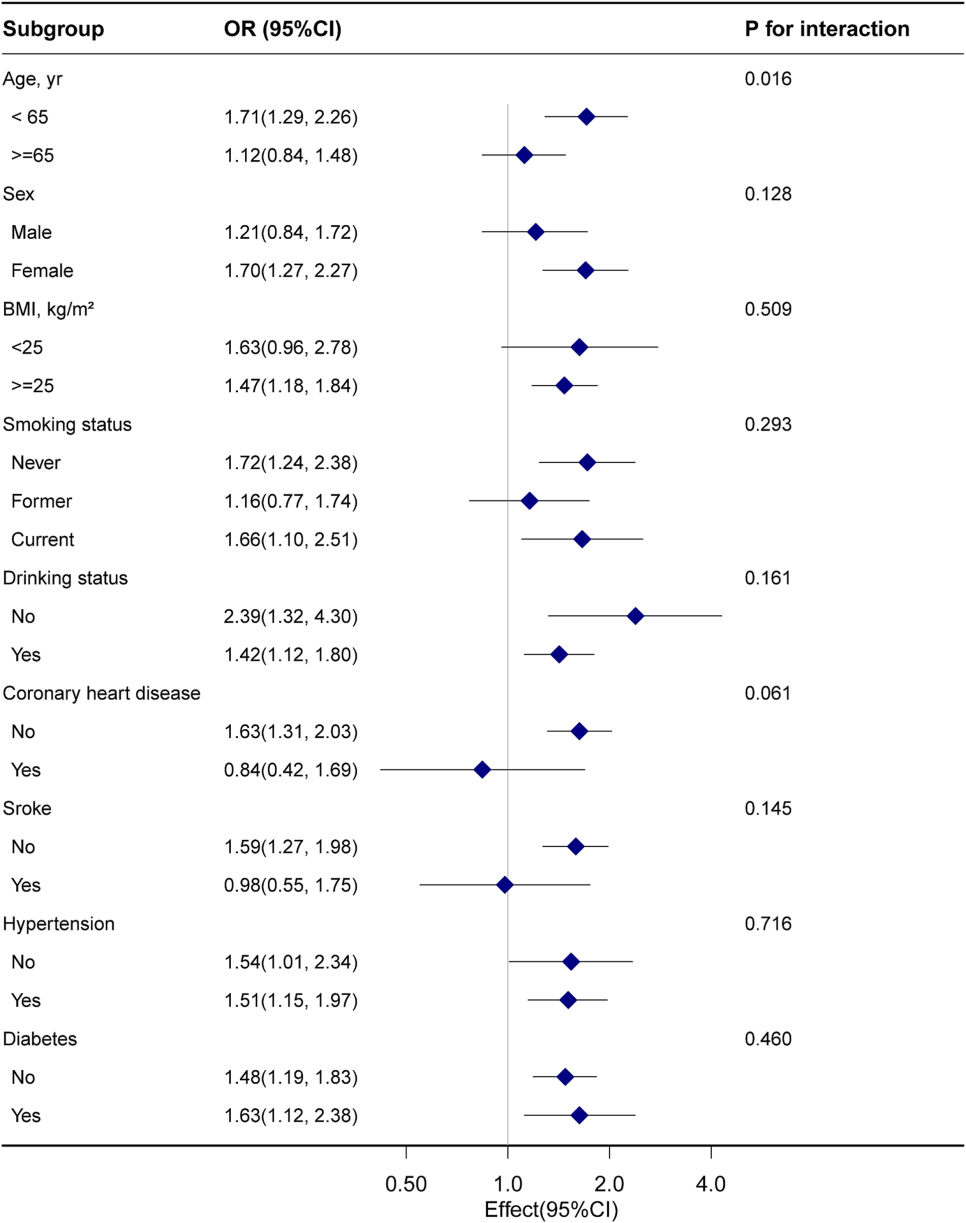
Subgroup analysis of rheumatoid arthritis and chronic obstructive pulmonary disease, weighted.

Subgroup analysis (Figure 2) showed that the association between RA and COPD remained consistent across various demographic and health-related factors. In the age subgroup analysis, the results indicated a *p* for the interaction of 0.016, but this was not statistically significant after Bonferroni correction for multiple testing. Overall, subgroup analyses showed consistent and robust associations between RA and COPD.

## 4 Discussion

In this nationally representative cross-sectional study, we found a positive association between RA and COPD, even after adjusting for sociodemographic factors, known risk factors, and comorbidities. Adults with RA had a 52% higher prevalence of COPD compared to those without RA, and subgroup analyses confirmed the stability of this association. Our results underline that patients with RA constitute a high-risk group warranting proactive respiratory monitoring and targeted preventive interventions to mitigate COPD-related morbidity in clinical practice.

In 2013, Nannini et al. (14) first reported that the incidence of COPD was significantly higher in patients with RA compared to those without RA. Subsequently, numerous studies have continued to report on the relationship between RA and COPD, with most suggesting that RA increases the risk of COPD (8, 19, 20, 23), consistent with our findings. Notably, a large prospective cohort study in Korea demonstrated that patients with RA had a higher risk of developing COPD compared to controls (aHR, 2.11; 95% CI, 1.96–2.28). Conversely, two cohort studies from Korea found a negative association between RA and COPD (21, 22), which researchers attributed to heterogeneity in study populations or definitions of COPD. This underscores the necessity of our nationally representative study, which provides new epidemiological information on the association between RA and COPD in US adults.

Several potential mechanisms could explain the association between RA and COPD. Firstly, RA is a systemic disease characterized by its impact on joints and multiple organ systems (10). It induces persistent systemic inflammation, which can result in significant immune dysfunction. A key feature of seropositive RA is the production of anti-citrullinated protein antibodies (ACPAs) (26), which can be generated in synovial sites and extra-articular locations, including the lung (15). ACPAs can form immune complexes and activate macrophages through binding with Fcgamma receptor IIa, leading to the release of pro-inflammatory cytokines such as TNF, IL-8, and IL-6 (16), which may cause damage to the pulmonary mucosa, airways, and interstitium. Additionally, *in vitro* studies have shown that ACPAs mediate neutrophil death and necrosis, promoting peripheral aberrant adaptive and innate immune responses via the externalization of citrullinated self-antigens and immunostimulatory molecules, establishing a perpetuating pathogenic mechanism (17). It has been suggested that the lungs themselves may be early targets of ACPA-induced damage, with chronic airway disease being a manifestation of ACPA-mediated pulmonary injury (12). Secondly, smoking poses a shared risk for the development of RA and COPD (27). Evidence supports a direct link between smoking and seropositive RA (28–31), suggesting that smoking may be the most critical exogenous factor for autoimmune development before the onset of RA. Smoking-induced oxidative stress (32), activation and dysregulation of innate and adaptive immune cells (33, 34), and epithelial cell renewal and regeneration after injury (35, 36) may be associated with the development of chronic lung disease in patients with RA. Therefore, smoking is likely a confounding factor for the association between RA and COPD. Our models adjusted for smoking status, and results indicated that RA remained positively associated with COPD even after accounting for smoking status. Notably, although we further included variables such as the SII in our multivariable model, the association between RA and COPD was only slightly attenuated. This minimal change does not necessarily suggest a negligible role of systemic inflammation; rather, it may reflect the limited sensitivity of SII in capturing the chronic low-grade inflammation and immune dysregulation that are characteristic of both RA and COPD. Given the limitations of the NHANES dataset, SII was the most representative inflammation-related marker available. Future studies are warranted to incorporate more specific immunological biomarkers, such as anti-citrullinated protein antibodies, cytokine profiles, or immune cell subtypes, to further elucidate the immunopathological mechanisms underlying the RA–COPD association.

The high prevalence of COPD in patients with RA poses substantial challenges, as current RA treatments do not have significant beneficial effects on COPD prevention or disease progression (12). Understanding the association between RA and COPD may provide opportunities for primary prevention of COPD in at-risk individuals, such as early patients with RA who smoke or smokers with a family history of RA. Nonetheless, additional studies are required to confirm the underlying cellular and molecular mechanisms of this association, aiding clinicians in closely monitoring disease progression and prognosis.

Given the potential shared immune and inflammatory pathways underlying RA and COPD, routine respiratory screening by rheumatologists is warranted in clinical practice. Patients with RA, particularly those presenting with respiratory symptoms or additional risk factors such as smoking history, should undergo timely pulmonary function assessments for early COPD detection and management. Incorporating COPD screening and management strategies into standard care for patients with RA could improve quality of life, slow disease progression, and reduce COPD-related hospitalizations and healthcare costs. Enhanced interdisciplinary collaboration between rheumatologists and pulmonologists is thus essential to optimize overall patient outcomes.

Longitudinal studies are warranted to clarify the temporal and causal relationship between RA and COPD. Biomarker validation will also be a critical future research direction; identifying biomarkers common to RA and COPD could enhance our understanding of shared pathological mechanisms and facilitate earlier diagnosis and individualized treatment strategies. Furthermore, genetic research exploring susceptibility loci may reveal how specific genotypes influence COPD risk in patients with RA. These studies will provide deeper insights into the RA-COPD association, thereby informing clinical intervention and public health strategies.

This study has several limitations. Firstly, the cross-sectional design limits the ability to infer causality between RA and COPD. Secondly, several variables—including the diagnosis of RA—were self-reported, introducing potential recall bias and misclassification. Third, despite adjusting for multiple covariates, residual confounding factors that were not accounted for in the analysis may still exist. Fourth, information on participants’ medication use was not available. Given that individuals with RA and COPD often receive long-term pharmacologic treatments, the lack of medication data could have influenced both disease status and the observed associations. Finally, these findings may not apply to different populations.

## 5 Conclusion

Rheumatoid arthritis is associated with COPD among U.S. adults, emphasizing the need for routine respiratory screening, timely pulmonary referral, and preventive interventions to reduce COPD-related morbidity and healthcare burden.

## Data Availability

The datasets presented in this study can be found in online repositories. The names of the repository/repositories and accession number(s) can be found below: the dataset generated and analyzed during the current study is available from the corresponding author upon reasonable request. Additionally, NHANES is a public dataset that can be freely accessed at http://www.cdc.gov/nchs/nhanes/.
