# Supplementary material for: Rheumatoid arthritis and chronic obstructive pulmonary disease in US adults: a cross-sectional analysis
Source: Front Med (Lausanne). 2025 May 23;12:1577180. doi: 10.3389/fmed.2025.1577180 (PMC12141009; doi:10.3389/fmed.2025.1577180)
Supplement: Supplementary file 1 [file Table_1.docx]

Table S1. Multivariable logistic regression analysis of the association between rheumatoid arthritis and chronic obstructive pulmonary disease by multiple imputations, weighted.

| Model | OR (95% CI) | *P*-value |
| --- | --- | --- |
| Crude Model | 2.93 (2.42~3.55) | <0.001 |
| Model 1^a^ | 2.06 (1.69~2.50) | <0.001 |
| Model 2^b^ | 1.66 (1.35~2.03) | <0.001 |
| Model 3^c^ | 1.64 (1.34~2.01) | <0.001 |

^a^Adjusted by sex and age; ^b^Adjusted by Model 1+ body mass index, race, smoking status, drinking status, marital status, education level, physical activity, poverty income ratio, diabetes, coronary heart disease, stroke, and hypertension; ^c^Adjusted by Model 2 + total cholesterol, high-density lipoprotein cholesterol, systemic immune inflammation index.
